# Systematic Violence Monitoring to Reduce Underreporting and to Better Inform Workplace Violence Prevention Among Health Care Workers: Before-and-After Prospective Study

**DOI:** 10.2196/47377

**Published:** 2023-11-13

**Authors:** Giovanni Veronesi, Marco Mario Ferrario, Emanuele Maria Giusti, Rossana Borchini, Lisa Cimmino, Monica Ghelli, Alberto Banfi, Alessandro Luoni, Benedetta Persechino, Cristina Di Tecco, Matteo Ronchetti, Francesco Gianfagna, Sara De Matteis, Gianluca Castelnuovo, Licia Iacoviello

**Affiliations:** 1 Research Center in Epidemiology and Preventive Medicine Department of Medicine and Surgery University of Insubria Varese Italy; 2 Occupational and Preventive Medicine Azienda Socio-Sanitaria Territoriale Lariana Como Italy; 3 Department of Occupational and Environmental Medicine, Epidemiology and Hygiene Italian Workers Compensations Authority (INAIL) Roma Italy; 4 Struttura Complessa Qualità, Risk Management e Accreditamento Azienda Socio-Sanitaria Territoriale dei Sette Laghi Varese Italy; 5 School of Specialization in Occupational Medicine University of Insubria Varese Italy; 6 Mediterranea Cardiocentro Napoli Italy; 7 Department of Health Sciences University of Milan Milan Italy; 8 Psychology Research Laboratory Istituto Auxologico Italiano Istituto di Ricovero e Cura a Carattere Scientifico Milano Italy; 9 Department of Psychology Catholic University of the Sacred Heart Milano Italy; 10 Department of Epidemiology and Prevention Istituto di Ricovero e Cura a Carattere Scientifico Neuromed Pozzilli Italy

**Keywords:** workplace, work, workers, worker, occupational health, safety, report, reporting, incident, abuse, health care workers, HCW, violence, surveillance, underreporting, risk, guidelines, incident report, Italy, prevention, workplace violence, hospital setting, assault, physical assaults, mental health, risk management

## Abstract

**Background:**

Monitoring workplace violence (WPV) against health care workers (HCWs) through incident reporting is crucial to drive prevention, but the actual implementation is spotty and experiences underreporting.

**Objective:**

This study aims to introduce a systematic WPV surveillance in 2 public referral hospitals in Italy and assess underreporting, WPV annual rates, and attributes “before” (2016-2020) and “after” its implementation (November 2021 to 2022).

**Methods:**

During 2016-2020, incident reporting was based on procedures and data collection forms that were neither standardized between hospitals nor specific for aggressions. We planned and implemented a standardized WPV surveillance based on (1) an incident report form for immediate and systematic event notification, adopting international standards for violence definitions; (2) second-level root cause analysis with a dedicated psychologist, assessing violence determinants and impacts and offering psychological counseling; (3) a web-based platform for centralized data collection; and (4) periodic training for workforce coordinators and newly hired workers. We used data from incident reports to estimate underreporting, defined as an observed-to-expected (from literature and the “before” period) WPV ratio less than 1, and the 12-month WPV rates (per 100 HCWs) in the “before” and “after” periods. During the latter period, we separately estimated WPV rates for first and recurrent events.

**Results:**

In the “before” period, the yearly observed-to-expected ratios were consistently below 1 and as low as 0.27, suggesting substantial violence underreporting of up to 73%. WPV annual rates declined in 1 hospital (from 1.92 in 2016 to 0.57 in 2020) and rose in the other (from 0.52 to 1.0), with the divergence being attributable to trends in underreporting. Available data were poorly informative to identify at-risk HCW subgroups. In the “after” period, the observed-to-expected ratio rose to 1.14 compared to literature and 1.91 compared to the “before” period, consistently in both hospitals. The 12-month WPV rate was 2.08 (95% CI 1.79-2.42; 1.52 and 2.35 in the 2 hospitals); one-fifth (0.41/2.08, 19.7%) was due to recurrences. Among HCWs, the youngest group (3.79; *P<*.001), nurses (3.19; *P<*.001), and male HCWs (2.62; *P=*.008) reported the highest rates. Emergency departments and psychiatric wards were the 2 areas at increased risk. Physical assaults were more likely in male than female HWCs (45/67, 67.2% vs 62/130, 47.7%; *P*=.01), but the latter experienced more mental health consequences (46/130, 35.4% vs 13/67, 19.4%; *P=*.02). Overall, 40.8% (53/130) of female HWCs recognized sociocultural (eg, linguistic or cultural) barriers as contributing factors for the aggression, and 30.8% (40/130) of WPV against female HCWs involved visitors as perpetrators.

**Conclusions:**

A systematic WPV surveillance reduced underreporting. The identification of high-risk workers and characterization of violence patterns and attributes can better inform priorities and contents of preventive policies. Our evaluation provides useful information for the large-scale implementation of standardized WPV-monitoring programs.

## Introduction

In 2019, the International Labour Organization (ILO) adopted the Violence and Harassment Convention to “promote and realize the right of everyone to a world of work free from violence and harassment” ([1], art 4). The European Foundation for the Improvement of Living and Working Conditions identifies the health and social work sector as the one with the highest prevalence of adverse social behaviors at work in Europe [2]. The official statistics from the Italian Workers Compensations Authority (INAIL) reported a mean of 2500 workplace violence (WPV) cases per year determining a work injury among health care workers (HCWs) over the 2016-2020 period; of these, 75% were directed toward women [3]. Due to the high prevalence and the consequences on the affected HCWs, WPV against HCW is a public health concern [4,5]. A meta-analysis of the studies published up to 2018—including mostly cross-sectional surveys—estimated for Europe a 12-month prevalence of exposure to nonphysical and physical WPV at 36.6% and 20.1%, respectively [4], with a high degree of heterogeneity across studies. These figures did not decline after the SARS-CoV-2 outbreak [5], exacerbating the impact of the pandemic on the mental health of HCWs [6]. In a more recent systematic review of Italian surveys, the 12-month prevalence of WPV ranged between 11.9% and 93.3% and between 27.5% and 50.3% for verbal and physical violence, respectively [7]. Such a large amount of heterogeneity reflects several methodological concerns, and it challenges the interpretation of results and their usability to guide preventive strategies [8]. First, most studies have been conducted on selected, high-risk wards and thus are poorly representative of the entire population. Second, the negative correlation between survey participation rate and reported violence prevalence [7] suggests the presence of self-selection bias. Finally, the retrospective evaluation of violence and the lack of a clear standardized definition of violence increase the risk of recall bias and measurement error [4,5,7,8].

Complementary to retrospective survey data, active monitoring and surveillance based upon systematic incident reporting is important not only to provide estimates of violence rates but also to investigate the circumstances in which WPV occurs, to identify violence determinants and attributes, and to quantify violence consequences on the assaulted workers [8-10]. Of note, these may also vary according to the assaulted HCW: for instance, evidence from Italy suggests the presence of sex-specific patterns in violence [11]. All this information is crucial to better inform prevention and mitigation strategies by health care organizations [8-11]. However, the implementation of such systems is still scanty and spotty [10]: to the best of our knowledge, standardized and regulatory-based violence monitoring programs implemented at a comprehensive regional- or state-level are present only in Australia [9] and in California [12,13]. The Lombardia Region—the most populated region in Italy with about 10 million inhabitants, located in the north of the country—published in 2019 new guidelines for WPV reporting, risk assessment, and management for regional public and private hospitals, to overcome the recognized WPV underreporting in ongoing registration systems [14]. Stemming from such document, our research group designed the study “Determinants of Violence against the Health care Workers” (*Determinanti Violenze Operatori Sanitari* [DeVOS]) to develop and implement a guideline-based incident report protocol for systematic WPV risk monitoring and management. With this paper, we aim to assess data completeness, WPV underreporting, and rates “before-and-after” the implementation of the new standard in 2 public hospitals (about 9000 employees) in the region. In addition, we report on violence attributes, contributing factors, and consequences and assess the presence of sex-specific patterns in these violence characteristics.

## Methods

### The DeVOS Study

The DeVOS study started in June 2020 in the only 2 publicly funded, referral hospitals (Azienda Socio-Sanitaria Territoriale [ASST]) serving the provinces of Varese (ASST Sette Laghi, hospital 1) and Como (ASST Lariana, hospital 2). Located in the Lombardia Region, the 2 provinces include about 1.5 million inhabitants, corresponding to 15% of the regional population. Each ASST includes hub-and-spoke hospitals, hospitals dedicated to mothers and children and rehabilitations, and outpatient clinics. To address the feasibility of our protocol in structures at different underlying WPV risks, we included all the hospitals comprising the ASSTs. The study had 4 main objectives: (1) to quantify WPV before the implementation of the new protocol (2016-2020) and to document the extent by which the available information on WPV episodes was compliant with the guidelines [14] across different hospitals; (2) to design and implement a new protocol for WPV reporting and management (Figure S1 in Multimedia Appendix 1), comprising of a standardized and easy-to-access incident report procedure gathering first information on violence attributes, consequences and contributing factors; of a root cause analysis for more in-depth assessment of violence determinants and impacts; and of a web-based platform for comprehensive event management and data collection; (3) to estimate WPV prevalence in the entire HCW population and in different subpopulations and wards after the implementation of the new standard; and (4) to assess the role of work organizational factors by estimating the association between turn-over, downsizing, sickness leaves, night shift working (defined according to a published method [15]), and WPV occurrence. The specific aims of this paper are related to objectives 1 to 3, whereas the role of organizational factors, as well as the psychological impact of violence, will be addressed in dedicated works. In accordance with regional guidelines [14] and international standards [16], WPV was defined as any form of verbal abuse, threats, physical assaults (to persons or things) and sexual harassment occurring at the workplace and perpetrated by hospital patients or visitors or hospital employees.

### WPV Reporting Before the Implementation of the Study Protocol (2016-2020)

During 2016-2020, incident reporting was based upon procedures and data collection forms that were neither standardized between hospitals nor specific for aggressions. The affected HCW notified the violence episode to the risk manager in 1 ASST and to the safety personnel in the other. The incident report data collection forms differed across hospitals and were also used for incidents other than WPV (eg, treatments adverse events, “near miss”), and several versions were adopted during the 5 years following changes in hospital’s organizations and managements. A root cause analysis was not implemented in either hospital.

### Development and Implementation of the Project’s WPV Reporting Protocol

Figure S1 in Multimedia Appendix 1 depicts the flow chart for the WPV reporting and management protocol of the DeVOS project. Assaulted HCWs were required to notify the WPV to the risk management office (hospital 1) or to the safety personnel (hospital 2) within 72 hours of occurrence by using a standardized, WPV-specific incident report data collection form, available on the hospitals’ intranet. The form was developed taking into account previous experience by the risk managers of the participating hospitals, regional guidelines [14], and existing literature on the topic [9,10,17,18], and it collected information on violence attributes (form, hospital ward, involved HCW, perpetrator, violence date and time, and environmental factors), consequences (physical, psychological, reactions, feelings during the aggression, and work injury), and contributing factors (sociocultural, structural, organizational, relational, and clinical). More details are in Table S1 in Multimedia Appendix 1. Most fields were not mutually exclusive, and the HCW could fulfill more than 1 choice. In the case of WPV involving multiple HCWs, the risk management office was required to check that each assaulted HCW filled in 1 incident report. HCWs signing the specific consent were further contacted by the psychologist for the root cause analysis, comprising of a structured interview including the Modified Overt Aggression Scale [19] and the Broset Violence Checklist [20]. Then, the HCW received to an email address of his or her choice a 1-time access link to the project web platform for a safe and easy completion of the questionnaires assessing the impact of the WPV event (presence of cognitive, emotional, and somatic symptoms [21]; the Maslach Burnout Inventory [22]; and the General Health Questionnaire-12 [23]) and psychosocial work conditions (the Italian version of the Health and Safety Executive’s Management Standards Indicator Tool [24]). These questionnaires were validated for use with Italian HCWs [21,24,25]. Furthermore, the psychologist contacted the HCW supervisor for a guided interview using a checklist on work content and context factors. During periodic training meetings with workforce coordinators, the risk management officers explained the new protocol and provided information on violence notification, field definitions on the incident report form to standardize data collection (including contributing factors), and the rationale and motivation for participation in the root cause analysis. The coordinators were then asked to instruct the HCW of their ward or unit. Additionally, in hospital 1, all newly hired HCWs were targeted by the same training. In both ASSTs, the new protocol became effective on November 1, 2021. One ASST (hospital 2) gradually introduced the protocol to the outpatient departments during the year 2022. Therefore, for that ASST, we included only the hub and the main spoke hospitals in the current analyses, corresponding to 71% of the workforce.

### Web-Based Platform for Data Collection and Risk Management

As part of the study, we developed a new web-based platform for centralized data collection, with customized access to data visualization or modification for risk management office personnel, the psychologist, and the data analyst. Due to ethical and confidentiality issues, we collected minimal sensitive information on the HCW and the aggressor. Furthermore, the easy visualization of questionnaire results by the psychologist allowed an immediate identification of high-risk workers for referral to supportive or mitigation resources.

### Ethical Considerations

The study received approval by the Ethical Committee of Insubria (IDs 82/2021 and 90/2021). Participants signed an informed consent on data confidentiality and protection and a separate informed consent to participate in the interview with the psychologist. The web-based platform for centralized data collection complied with the European Union legislation on data protection. The platform applied a complete anonymization of personal data, allowing the tracing of repeated events on the same worker. HCWs received no compensation for their participation in the study.

### Statistical Analysis

To be consistent with the literature mostly reporting WPV prevalence for a 12-month interval, for each year in the “before” study period (2016-2020) and by hospital, we estimated the 12-month WPV rate (per 100 workers) as the ratio between the number of reported WPV in the year and the number of HCWs in the payroll administrative records at the beginning of each year. The 95% CI for the yearly rate was estimated using the exact binomial distribution [26]. We estimated underreporting as the ratio between the observed and expected number of violent incidents. For the latter, we used the WPV rate estimated in 2015-2017 in a large public University hospital in Northern Italy by Viottini and colleagues [27] as 210 observed incidents each year over a population of 10,970 HCWs, along with the number of HCWs, again from payroll administrative records. The 95% CI for the ratio was estimated from the Poisson exact method [26]. To date, we completed the first 14 months since the implementation of the new reporting standard (November 1, 2021 to December 31, 2022). For the entire period, we reported on data completeness as missing data prevalence on the incident report data collection forms. To describe the violence attributes, we first assessed the co-occurrence of different types of violence in the same episode, by reporting percentages and mean number of violence types by episode. Then, we reported the distribution of major violence characteristics, consequences, and contributing factors, in the overall sample and by sex of the assaulted HCWs, to identify the presence of sex-related patterns. The association between violence characteristics and sex was formally tested through chi-square tests. The analyses of WPV underreporting and rate were restricted to a 12-month period (year 2022, n=166 WPV) adopting the same methods described above for the “before” period and using the number of HCWs in the payroll records at the beginning of the implementation of the new standard. The overall rate was then broken down as the sum of 2 mutually exclusive components: 1 being the rate of the first violent episode and the second being the rate of recurrent violent episodes on the same worker. Statistical analyses were performed using SAS software (version 9.4; SAS Institute Inc).

## Results

### Data Completeness and WPV Rate in the “Before” Period

**Figure 1:**
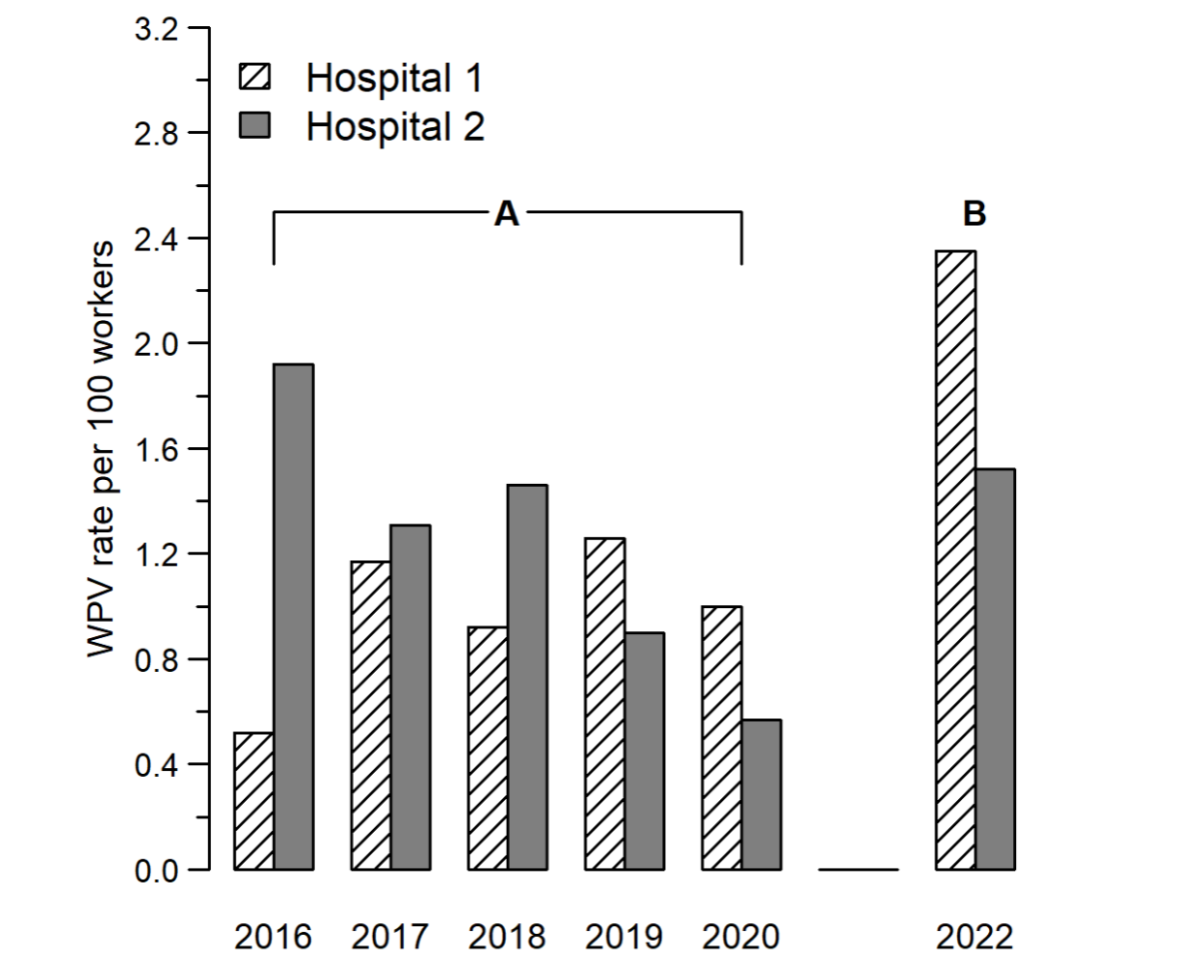
Twelve-month WPV rate observed in the study hospitals, before (period A) and after (period B) the implementation of the study protocol on incident report. The study protocol on incident report was effective since November 1, 2021. The WPV rate (per 100 health care workers) is the number of observed WPV incidents divided by the number of health care workers at the beginning of each year. WPV: workplace violence.

Between 2016 and 2020, a total of 400 WPV reports were notified in the 2 study hospitals. Data completeness was scanty: in 160 (40%) of the reports, the affected HCW was not identified (eg, replaced by a “generic” description of job title or by expressions such as “everyone present”), and when identifiable, in 70.8% (170/240) of reports, there was at least 1 missing information on age, sex, or job title. This prevented us from providing specific prevalence estimates by subgroups. Similarly, environmental details, consequences, and contributing factors were not available in 59% (236/400), 18% (72/400), and 50% (200/400) of reports. Yearly WPV rates were diverging between the 2 study hospitals, increasing from 0.52 (95% CI 0.34-0.77) per 100 HCWs to 1.0 (95% CI 0.75-1.31) in 1 hospital and declining from 1.92 (95% CI 1.42-2.54) to 0.57 (95% CI 0.32-0.93) in the other (Figure 1A). The yearly observed to expected ratio was significantly below 1 for most years, suggesting substantial (up to 73%) underreporting. In particular, the ratio from 2016 to 2020 changed from 0.27 to 0.52 (eg, reduction in underreporting) in the hospital with an increasing WPV rate and from 1.0 to 0.30 (eg, increase in underreporting) in the other.

### Data Completeness and WPV Rate in the “After” Period

During the first 14 months of implementation of the study standard, a total number of 205 WPV were reported, half (50%) of them on the same day of occurrence, and 75% within the first 72 hours. Data completeness was optimal: only in 2% (4/205) of reports the affected HCW was not identified, and when identifiable, only in another 2% (4/201) there was at least 1 missing information on demographics or job title information (Table S1 in Multimedia Appendix 1). The observed to expected ratio was 1.91 (95% CI 1.63-2.21) as compared to the “before” period in the same hospitals, which was consistent in the 2 hospitals (1.96 and 1.77). When using the reference rate from literature to estimate the expected number of WPVs, the observed to expected ratio was 1.14 (95% CI 0.98-1.32), being slightly lower in hospital 2 where the first notification was to safety personnel (0.83, 95% CI 0.60-1.13) rather than to the risk management office (1.28, 95% CI 1.07-1.52) as in hospital 1. The 12-month WPV rate was 2.08 (95% CI 1.79-2.42) per 100 HCWs (Table 1), being slightly lower in hospital 2 than in hospital 1 (1.52 vs 2.35; *P*=.02; Figure 1B). WPV rate (Table 1) declined with age, with the youngest group being at the highest risk (*P*<.001); it was higher in male than female HCWs (2.82 vs 1.83; *P*=.008); and it was more than doubled in nurses (3.49; 95% CI 2.93-4.15), as compared to nurse assistants (1.74) and physicians (1.08; *P*<.001). Finally, psychiatric wards (14.3) and emergency departments (13.3) were at higher WPV rates than the remaining wards; about one-fifth (0.41/2.08, 19.7%) of the rates are due to recurrences. Analyses by hospital (Table S2 in Multimedia Appendix 1) suggested higher rates in the youngest group in hospital 1 and in psychiatric wards in hospital 2, whereas associations by sex and job title were homogeneous across the hospitals.

**Table 1:** Twelve-month WPV^a^ rate (with 95% CI) for the year 2022 by affected HCW^b^ demographic and work characteristics^c^.

Characteristics		HCWs (n=7982), n (%)	WPV (n=166), n (%)^d^	12-month WPV rate, (95% CI)				
				First WPV^e^	Recurrent WPV^f^	Overall WPV^g^	*P* value^h^	
All WPV		7982 (100)	166 (100)	1.67 (1.41-1.97)	0.41 (0.29-0.58)	2.08 (1.79-2.42)	—^i^	
**Age group (years)**								<.001
	<30	829 (10.4)	33 (19.9)	2.90 (1.95-4.28)	1.09 (0.57-2.07)	3.98 (2.85-5.54)		
	30-50	3652 (45.8)	88 (53)	1.86 (1.47-2.36)	0.55 (0.35-0.85)	2.41 (1.96-2.96)		
	>50	3501 (43.9)	45 (27.1)	1.17 (0.86-1.59)	0.11 (0.04-0.30)	1.29 (0.96-1.72)		
**Sex**								.008
	Female	5960 (74.7)	109 (65.7)	1.51 (1.23-1.85)	0.32 (0.20-0.50)	1.83 (1.52-2.20)		
	Male	2022 (25.3)	57 (34.3)	2.13 (1.58-2.86)	0.69 (0.41-1.17)	2.82 (2.18-3.64)		
**Job title**								<.001
	Physician	1296 (16.2)	14 (8.4)	0.93 (0.53-1.62)	0.15 (0.04-0.61)	1.08 (0.64-1.82)		
	Nurse	3498 (43.8)	122 (73.5)	2.69 (2.20-3.28)	0.80 (0.55-1.12)	3.49 (2.93-4.15)		
	Nurse assistant	1149 (14.4)	20 (12.0)	1.48 (0.92-2.37)	0.26 (0.08-0.81)	1.74 (1.13-2.68)		
	Health care technician	833 (10.4)	2 (1.21)	0.24 (0.06-0.95)	—	0.24 (0.06-0.95)		
	Administrative clerk	824 (10.3)	1 (0.60)	0.12 (0.002-0.86)	—	0.12 (0.002-0.86)		
	Other	382 (4.8)	7 (4.2)	1.83 (0.88-3.79)	—	1.83 (0.88-3.79)		
**Hospital ward**								<.001
	Psychiatry and mental health departments	322 (4)	46 (27.7)	10.6 (7.64-14.4)	3.73 (2.13-6.44)	14.3 (10.9-18.6)		
	Emergency department	592 (7.4)	79 (47.6)	10.1 (7.95-12.8)	3.21 (2.06-4.98)	13.3 (10.8-16.3)		
	Other wards	7068 (88.5)	41 (24.7)	0.55 (0.40-0.75)	0.03 (0.001-0.11)	0.58 (0.43-0.79)		

^a^WPV: workplace violence.

^b^HCW: health care worker.

^c^Twelve-month rate is the ratio between the number of WPV and the number of HCWs, per 100 HCWs.

^d^Excluding 8 WPVs without data on age, sex, and job title of the affected HCW.

^e^First WPV: number of first WPVs in the 12-month period.

^f^Recurrent WPV: number of WPVs from the second case onward, in the 12-month period.

^g^The overall violence rate is the sum of first and recurrent violence rates.

^h^Wald chi-square test on the overall violence reporting rate, with a number of degrees of freedom equal to the number of classes in the independent variable – 1.

^i^Not applicable (no groups to be compared).

### Violence Attributes and Patterns in the “After” Period

Table 2 reports the co-occurrence of violence forms in the incident reports. Verbal abuse was the largest majority (186/205, 90.7%) Of these, verbal abuse was the only form of violence in 26 (14%) out of 186 incidents, whereas 134 (72%) also reported threats, 98 (52.7%) reported physical assaults, and 15 (8%) reported sexual harassment. The mean number of reported violence forms was 2.33. We report similar figures for the other violence forms. Table 3 reports the distribution of major violence characteristics and risk factors, by sex of the assaulted HCW. Male HCWs were more likely than female HCWs to be subject to physical assault (45/67, 67.2% vs 62/130, 47.7%; *P*=.01). Either alone or with a patient, visitors were more likely to assault female than male HCWs (40/130, 30.7% vs 8/67, 12%; *P*=.02). No sex-related differences were observed on violence time or location. Female HCWs were less likely than male HCWs to report a work injury to the insurance authority (4/130, 3.1% vs 15/67, 22.4%; *P*<.001) and were more likely than men to report psychological consequences (46/130, 35.4% vs 13/67, 19.4%; *P*=.02) and life-threatening feelings during the aggression (23/130, 17.7% vs 5/67, 7.5%; *P*=.05). Finally, 47.8% (32/67) of WPV in male HCWs was related to the clinical conditions of the perpetrator, which is greater than that in female HCWs (40/130, 30.8%; *P*=.02). In female HCWs, the single most frequently reported factor was the sociocultural one (eg, related to linguistic or cultural barriers or behaviors; 53/130, 40.8%). Of note, structural factors including safety (the lack of barriers, alarm systems, or escape or poor lightning) were among the least prevalent identified contributing factors, both in men and in women.

**Table 2:** Co-occurrence of different types of violence observed in the “after” period.

Type of violence (number of reports)	Co-occurrence, n (%)^a^					No co-occurrence, n (%)^b^		Number of violence types in incident reports, mean
	VA^c^	T^d^	PA^e^	SH^f^				
VA (n=186)	—^g^	134 (72)	98 (52.7)	15 (8)	26 (14)		2.33	
T (n=139)	134 (96.4)	—	77 (55.4)	12 (8.6)	2 (1.4)		2.60	
PA (n=115)	98 (85.3)	77(67)	—	10 (8.7)	14 (12.2)		2.61	
SH (n=15)	15 (100)	12 (80)	10 (66.7)	—	0 (0)		3.46	

^a^Percent of incident reports with the row type of violence that also reports the column type.

^b^Percent of incident reports with only the row type of violence.

^c^VA: verbal abuse.

^d^T: threat.

^e^PA: physical assault.

^f^SH sexual harassment.

^g^Not relevant.

**Table 3:** Distribution of major violence attributes, consequences, and contributing factors, by sex of the assaulted HCW^a^, in the “after” period.

Attributes			All WPV^b^ (n=197), n (%)		WPV against female HCWs (n=130), n (%)		WPV against male HCWs (n=67), n (%)		*P* values	
**Type of violence^c^**										
	Verbal abuse	179 (90.7)		120 (92.3)		59 (88.1)		.33		
	Threat	132 (67.0)		82 (63.1)		50 (74.6)		.10		
	Physical assault	107 (54.3)		62 (47.7)		45 (67.2)		.01		
	Sexual harassment	14 (7.1)		8 (6.2)		6 (9.0)		.47		
**Perpetrator**										.02
	Patient only	138 (70.1)		82 (63.1)		56 (83.6)				
	Visitor only	38 (19.3)		31 (23.9)		7 (10.5)				
	Patient and visitor	10 (5.1)		9 (6.8)		1 (1.5)				
	Coworker	11 (5.6)		8 (6.9)		3 (4.5)				
Violence during a night shift			68 (34.7)		43 (33.3)		25 (37.3)		.58	
**When during the work shift**										.73
	Beginning	25 (12.8)		16 (12.4)		9 (13.4)				
	During	162 (82.7)		106 (81.2)		56 (83.6)				
	End	9 (4.6)		7 (5.4)		2 (3.0)				
**Location^c^**										
	Patient’s bedroom	45 (22.8)		29 (22.3)		16 (23.9)		.80		
	Waiting examination room	108 (54.8)		71 (54.6)		37 (55.2)		.94		
	Communal location^d^	54 (27.4)		35 (26.9)		19 (28.4)		.83		
	External areas	20 (10.2)		15 (11.5)		5 (7.5)		.37		
	Other^e^	6 (3.1)		5 (3.9)		1 (1.5)		.36		
**Consequences^c^**										
	Physical	39 (19.8)		22 (16.9)		17 (25.4)		.16		
	Psychological	59 (30.0)		46 (35.4)		13 (19.4)		.02		
	Life-threatening feeling	28 (14.2)		23 (17.7)		5 (7.5)		.05		
Work injury report^f^			19 (9.6)		4 (3.1)		15 (22.4)		<.001	
**Contributing factors^c,g^**										
	Sociocultural	80 (40.6)		53 (40.8)		27 (40.3)		.95		
	Structural	30 (15.2)		18 (13.9)		12 (17.9)		.45		
	Organizational	60 (30.5)		41 (31.5)		19 (28.4)		.65		
	Relational	55 (27.9)		37 (28.5)		18 (26.9)		.81		
	Clinical	72 (36.6)		40 (30.8)		32 (47.8)		.02		
	Other	18 (9.1)		15 (11.5)		3 (4.5)		.10		
	Not identified	28 (14.2)		18 (13.9)		10 (14.9)		.84		

^a^HCW: health care worker.

^b^WPV: workplace violence.

^c^More than 1 answer was possible, so the total does not sum up to 100%.

^d^For example, corridors, stairs, and elevators.

^e^Including web-based violence via email or telephone.

^f^Violence determining a work injury must be reported to the Italian Workers Compensations Authority.

^g^Sociocultural (eg, linguistic barriers, behavior conditioned by cultural elements such as education or country of origin, the inadequacy of social behaviors); structural (eg, lack of barriers/alarm systems/escape, poor lightning); organizational (eg, related to work organization); relational (eg, related to HCW experience and communication abilities); and clinical (eg, related to the conditions of the perpetrator, including psychiatric disorders and substance abuse).

## Discussion

The implementation of the ILO convention [1] and its subsequent ratification in state members, including Italy (Law n. 4 of January 15, 2021), calls for a systematic and standardized registration of WPV in HCWs implemented on a large scale to inform prevention, protection, and enforcement actions [7]. In Italy so far, national statistics cover only more severe cases, such as those exiting in a work injury [3], whereas academic data obtained from convenient samples and retrospective investigations exhibit significant heterogeneity, rendering them uninformative for guiding prevention efforts effectively [7]. This study documents underreporting and WPV prevalence estimates in 2 large public referral hospitals before and after the implementation of a standardized, guidelines-based program for systematic violence monitoring. Similar experiences [9,12,13] have not yet documented such temporal changes. WPV data in HCWs are affected by a substantial amount of underreporting [28,29], up to 80% to 90% according to 1 US study [29]. In line with this previous knowledge, in the 2016-2020 period, we estimated a yearly observed to expected ratio mostly below 1 in the 2 study hospitals and as low as 0.27, corresponding to 73% underreporting toward expected prevalence from the literature [27]. Such a large amount of underreporting hampers a meaningful interpretation and comparison of time trends in violence prevalence between the structures. After the implementation of the new standard, the 12-month observed to expected ratio increased to be larger than 1 as compared to the same benchmark and almost 2 as compared to the “before” period in the same hospitals. In addition, only 9.8% (20/205) of the reported WPV determined a work injury, suggesting that we were fairly able to detect less serious incidents in which “no one was hurt,” which are generally overlooked [30]. Finally, although verbal abuse was present in 90.7% (186/205) of reports, the affected HCWs were able to describe and report the complex co-occurrence of violence forms in the same episode. However, in the study hospital in which the first notification was to safety personnel, rather than to the risk management office, we found a lower observed to expected ratio, a lower 12-month overall WPV rate but with a higher peak in psychiatric wards, where episodes are more likely to be related to the psychiatric conditions of the perpetrator. Based on these findings, notification to dedicated personnel is recommended in future applications to enhance violence reporting.

Several reasons have been advocated for WPV underreporting, including the feeling that violence is “part of the job” [31], the lack of supervisor or coworker support, the fear of blame, and the belief that reporting would not lead to positive changes [29]. We may speculate that our protocol could have contrasted some of these barriers, through the introduction of a new reporting standard specifically dedicated to WPV; the periodic training of the new procedure with the workforce and newly hired workers; the easiness of reporting by the HCW along with a close support contact with the risk management office during the first 72 hours since the episode; the availability of a dedicated psychologist for counseling, also outside the HCW working hours; and the periodic sharing of reporting with workers’ safety representatives for health. These aspects, specifically related to our protocol, might have reinforced the positive impact on violence reporting related to increased awareness of the issue due to the perception that the institution was “giving attention to it.” Taken together, these put into light the crucial role of a participative approach to the management of WPV that, starting from standardized and systematic data collection, requires the inclusion and active involvement of the organization, workers, and occupational health figures (risk management, occupational physicians, psychologist, and workers’ safety representatives).

The 12-month violence rates we estimated should be compared with caution with those from survey studies, since ours are referring to the entire HCW population and are based on incident reporting rather than on recollection. We add important pieces of information that can help to identify high-risk workers. First, while confirming the highest violence rates for psychiatric wards, emergency departments, and nurses, we add the notion that about 25% of their rate is due to the reoccurrence of aggressions on the same workers, as previously observed in Italy [27,32], Norway [33], and Denmark [34]. As current knowledge on the factors related to multiple aggressions is so far limited [33,34], future analyses are required to elucidate the role of personal and work-related characteristics on multiple aggressions. Second, in identifying high-risk subpopulations, it is important to consider that age, sex, and job title might have an unbalanced distribution in the specific health care workforce. Although in our data the large majority (66%, 130/167) of incident reports involved female HCWs, confirming institutional data [3], these do not appear to be at higher WPV risk as compared to male HCWs once their disproportional number in the workforce has been considered, as in other prospective studies [33,35]. Finally, our data can be used to well-characterize patterns in WPV attributes and consequences related to HCW’s characteristics, of which the sex-related one is an exemplification. To this extent, we confirmed the highest prevalence of physical violence in male HCWs recently observed in Italy [11], at the same time expanding knowledge by reporting sex-specific patterns in consequences, contributing factors, and conditions of the perpetrator. Taken together, this information can both guide priorities of interventions and a better design of the contents of primary, secondary, and tertiary prevention policies [36,37]. Systematic incident reporting is important to provide a “time zero” to evaluate the efficacy of future interventions.

Study limitations include the short time period of implementation of the new standard; since it is still in use by the 2 hospitals, we will be able to monitor in future reports its sustainability over time. Our study cannot document changes in WPV prevalence before and after the SARS-CoV-2 pandemic outbreak, given that the change in the reporting system became effective only at the end of 2021. Our underreporting metric is based upon a violence rate derived from literature, but assuming it remained constant during the study periods (2016-2020 and 2022). On the other hand, due to the substantial heterogeneity documented in the introduction, the validity of the metric depends upon the choice of a comparable benchmark for study design, period, and setting, since violence prevalence in Italy is also heterogeneous across public versus private hospitals [32] and country area [38]. The study we chose [27] satisfies most of these requirements. In addition, we used the annual count of HCWs to mitigate the impact on the expected number of incidents due to changes in the workforce. Then, our WPV rates can provide an updated benchmark for assessing underreporting in Italy, using a similar observed-to-expected metric. Our study protocol was implemented and tested in 2 large public general hospitals, each with a complex organizational structure including hub-and-spoke hospitals, as well as rehabilitation and outpatient clinics. We adopted recognized standards for violence definition, attributes, consequences, and determinants [10], to enhance the comparison of our findings with other studies.

In conclusion, a guidelines-based protocol can mitigate the underreporting of violence episodes against HCWs and provide accurate information to identify high-risk workers and describe violence attributes and patterns. The standardization across hospitals can better inform priorities and contents of preventive policies, at both a local and a large scale. To this extent, our evaluation can provide useful information for large-scale implementation of guidelines-based monitoring programs, as well as in other contexts.

## Appendix

Multimedia Appendix 1 Study flow chart, data fields in the report form and prevalence of missing data, and workplace violence rates by study hospital.

## Abbreviations

ASST: Azienda Socio-Sanitaria Territoriale
DeVOS: Determinanti Violenze Operatori Sanitari (Determinants of Violence against the Health care Workers)
HCW: health care worker
ILO: International Labour Organization
INAIL: Italian Workers Compensations Authority
WPV: workplace violence

## Data Availability

The data sets generated and analyzed during this study are not publicly available due to restrictions imposed by the informed consent obtained for this study and by the legislation of the European Union, but they can be made available from the corresponding author on reasonable request. Approval by each management of the Azienda Socio-Sanitaria Territoriale that participated in the study will be required for the release of the data.
