# Supplementary material for: Systematic Violence Monitoring to Reduce Underreporting and to Better Inform Workplace Violence Prevention Among Health Care Workers: Before-and-After Prospective Study
Source: JMIR Public Health Surveill. 2023 Nov 13;9:e47377. doi: 10.2196/47377 (PMC10682923; doi:10.2196/47377)
Supplement: Multimedia Appendix 1 [file publichealth_v9i1e47377_app1.docx]

**Multimedia Appendix Tables and Figures**

**Figure S1.** Flow chart of the Determinanti Violenze Operatori Sanitari project’s workplace violence reporting protocol. WPV: workplace violence; HCW: health care worker.


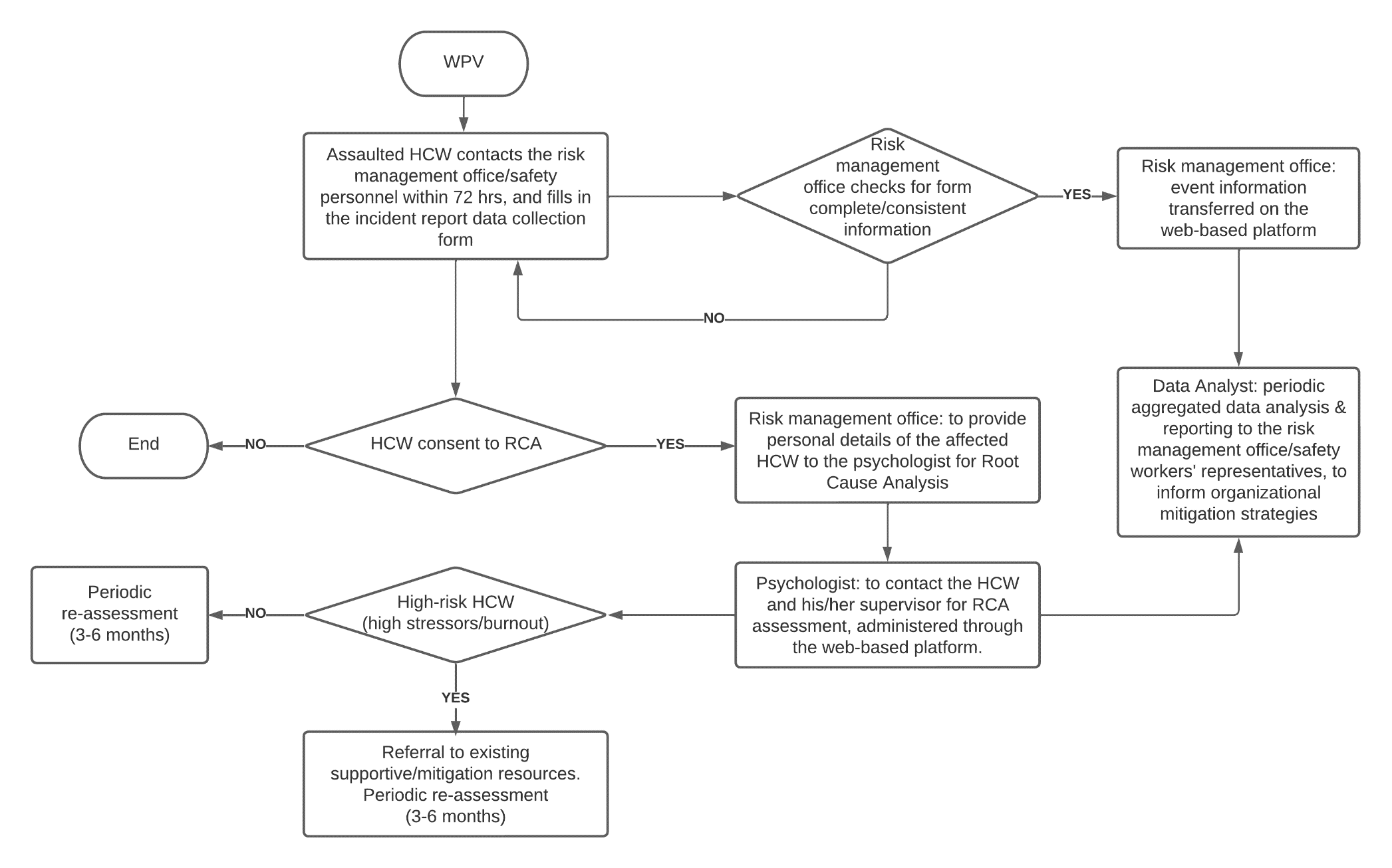


**Table S1.** Data fields included in the incident report data collection form, and prevalence of missing data in the first 14 months since the introduction of the study incident report system.

| Data fields | Description | Number of WPV^a^ | Missing data, n (%) |
| --- | --- | --- | --- |
| Violence form | Verbal abuse, threat, physical assault, sexual harassment | 205 | 0 (0.0) |
| Hospital ward | Identification of the hospital ward | 205 | 0 (0.0) |
| Identification of the affected HCW^b^ | Individual vs. generic description of the involved HCWs | 205 | 4 (1.96) |
| Characteristics of the affected HCW | Age, gender, job title^c^ | 201 | 4 (2.0) |
| Perpetrator | Patient/visitor (type-II), coworker (type-III) | 205 | 0 (0.0) |
| Date and time of violence | Night violence, time during the work shift | 205 | 1 (0.5) |
| Environmental factors | Location, isolated/alone | 205 | 0 (0.0) |
| Consequences on the affected HCW | Physical and psychological consequences | 205 | 0 (0.0) |
| Contributing factors^d^ | Sociocultural, structural, organizational, relational, clinical condition of the perpetrator | 205 | 0 (0.0) |

^a^WPV: workplace violence.

^b^HCW: health care worker.

^c^Amongst WPV reports which allowed the identification of the affected HCW (as reported in the third line of the table).

^d^Contributing factors: sociocultural (eg, linguistic barriers, behavior conditioned by cultural elements such as education or country of origin, inadequacy of social behaviors); structural (eg, lack of barriers/alarm systems/escape, poor lightning); organizational (eg, work stress, workload, lack of personnel, long waiting time, room overcrowding); relational (eg, HCW communication abilities and working seniority); and clinical (characteristics/clinical conditions of the perpetrator, including presence of psychiatric disorders and substance abuse).

**Table S2.** WPV^a^ rate of 12-months (with 95% CI) for the year 2022 by study hospitals^b^.

|  | | 12-month WPV rate (95% CI) | | | |
| --- | --- | --- | --- | --- | --- |
|  | | Overall | Hospital 1 | Hospital 2 | *P* values^c^ |
|  | |  |  |  |  |
| All WPV | | 2.08 (1.79-2.42) | 2.35 (1.98-2.79) | 1.52 (1.11-2.07) | — |
| **Age class in years** | | | | | <.001 |
|  | <30 | 3.98 (2.85-5.54) | 5.35 (3.74-7.60) | 1.39 (0.52-3.65) |  |
|  | 30-50 | 2.41 (1.96-2.96) | 3.00 (2.39-3.77) | 1.28 (0.78-2.07) |  |
|  | >50 | 1.29 (0.96-1.72) | 1.05 (0.72-1.54) | 1.85 (1.18-2.87) |  |
| **Sex** | | | | | .40 |
|  | Women | 1.83 (1.52-2.20) | 2.12 (1.72-0.26) | 1.19 (0.78-1.80) |  |
|  | Men | 2.82 (2.18-3.64) | 3.07 (2.26-4.16) | 2.36 (1.48-3.77) |  |
| **Job title** | | | | | .14 |
|  | Physician | 1.08 (0.64-1.82) | 1.49 (0.87-2.55) | 0.24 (0.03-1.65) |  |
|  | Nurse | 3.49 (2.93-4.15) | 4.10 (3.38-4.97) | 4.67 (1.86-11.2) |  |
|  | Nurse-assistant | 1.74 (1.13-2.68) | 1.67 (0.97-2.85) | 0.26 (0.03-2.13) |  |
| **Hospital ward** | | | | | <.001 |
|  | Psychiatry and mental-health department | 14.3 (10.9-18.6) | 11.7 (8.4-16.2) | 25.9 (16.2-38.6) |  |
|  | Emergency department | 13.3 (10.8-16.3) | 16.4 (12.9-20.5) | 8.44 (5.5-12.9) |  |
|  | Other wards | 0.58 (0.43-0.79) | 0.75 (0.54-1.04) | 0.22 (0.10-0.52) |  |

^a^WPV: workplace violence.

^b^12-month rate as the ratio between the number of WPV and the number of HCW, per 100 healthcare workers.

^c^Wald chi-square test for homogeneity of effects between the study hospitals, from logistic regression models with a hospital*HCW feature interaction.
